# Atypical neuroimaging characteristics of hemophagocytic lymphohistiocytosis in infants: a case series of hemorrhagic brain lesions in the deep grey matter

**DOI:** 10.1007/s00234-020-02595-6

**Published:** 2020-11-06

**Authors:** Neda Pak, Anseh Selehnia, Maayke A. W. Hunfeld, Maarten H. Lequin, Rinze F. Neuteboom, Andrica C. H. de Vries, Andre A. Kroon, Marjolein H. G. Dremmen

**Affiliations:** 1 Department of Radiology & Nuclear Medicine, Erasmus Medical Center—Sophia Children’s Hospital, P.O. Box 2060, CB 3000 Rotterdam, The Netherlands; 2 Department of Radiology, Tehran University of Medical Sciences—Children’s Medical Center, Tehran, Iran; 3 Department of Neurology, Erasmus Medical Center—Sophia Children’s Hospital, Rotterdam, The Netherlands; 4 Department of Radiology, University Medical Center Utrecht—Wilhelmina Children’s Hospital, Utrecht, The Netherlands; 5 Department of Pediatrics, Erasmus Medical Center—Sophia Children’s Hospital, Rotterdam, The Netherlands; 6 Department of Neonatology, Erasmus Medical Center—Sophia Children’s Hospital, Rotterdam, The Netherlands

**Keywords:** Hemophagocytic lymphohistiocytosis, MRI brain, Infant

## Abstract

Hemophagocytic lymphohistiocytosis (HLH) is a rare multisystem condition associated with uncontrolled overproduction and infiltration of lymphocytes and histiocytes predominantly in liver, lymph nodes, spleen, and central nervous system. Neuroimaging findings on MRI are fairly nonspecific and classically include periventricular white matter signal abnormalities and diffuse atrophy. Focal parenchymal lesions may demonstrate post contrast ring or nodular enhancement and calcification. However, the MR imaging characteristics can be highly variable. Here, we present two cases of HLH in infants with multiple hemorrhagic lesions mostly depicted in both thalami and basal ganglia regions. Thalamic, basal ganglia, and brain stem involvement with hemorrhagic changes in HLH are rarely described in literature. Early diagnosis of HLH may be lifesaving. Awareness of the disease is necessary to investigate its characteristic findings and avoiding a delay in diagnosis.

## Introduction

Hemophagocytic lymphohistiocytosis (HLH) is an uncommon life-threatening hematologic disease more prevalent in children than in adults. The factual incidence is unknown due to difficulties in identifying the disease. An estimated prevalence of pediatric HLH cases is 1.07/100,000 [1]. The disease is characterized by uncontrolled overproduction and infiltration of lymphocytes and histiocytes. HLH typically involves multiple organ systems and predominantly affects the liver, spleen, lymph nodes, and the central nervous system (CNS) [1]. The primary form of HLH is familial (autosomal recessive or X-linked) and occurs in infants with predisposing inherited immune deficiencies. In addition, “acquired” HLH can be seen in older children secondary due to infection, autoimmune disease, immunosuppression, and malignancies [1, 2]. The diagnosis of HLH requires five out of eight defined clinical and laboratory criteria to be fulfilled as defined by the Histiocyte Society: fever, splenomegaly, cytopenia, hypertriglyceridemia, hemophagocytosis, low or absent NK-cell activity, elevated ferritin, and elevated soluble CD25 [1]. CNS involvement is frequent and is one of the major causes of mortality in HLH patients [1, 2]. Variable neurologic manifestations such as seizures, cranial nerve palsies, ataxia, nystagmus, irritability, depressed consciousness level, and hypotonia have been reported [3].

Neuroimaging findings typically are not very specific and include periventricular white matter signal abnormalities and ring or nodular enhancement of focal parenchymal lesions on MR imaging [4]. Involvement of deep gray matter and hemorrhagic lesions are rare findings in HLH disease [5].

This review presents two cases of HLH with multiple hemorrhagic lesions mostly depicted in both thalami and basal ganglia region.

## Case 1

A 3-month-old girl presented with poor feeding, vomiting, altered state of consciousness, and pallor. Her mother passed away 2 weeks after (a non-complicated) delivery, because of pulmonary edema followed by multi-organ failure (no autopsy performed). Physical exam revealed small head circumference (38.5 cm (−1.6 SD)) and hepatosplenomegaly. The eyes were deviated to the left with a nystagmus, repeated protrusion of the tongue was seen, and there was axial hypotonia. Laboratory findings showed anemia, thrombocytopenia, elevated levels of ferritin, triglyceride, and soluble interleukin-2. On abdominal ultrasound, hepatosplenomegaly and nephromegaly were present. Cranial ultrasound demonstrated increased echogenicity of the basal ganglia.

**Fig. 1:**
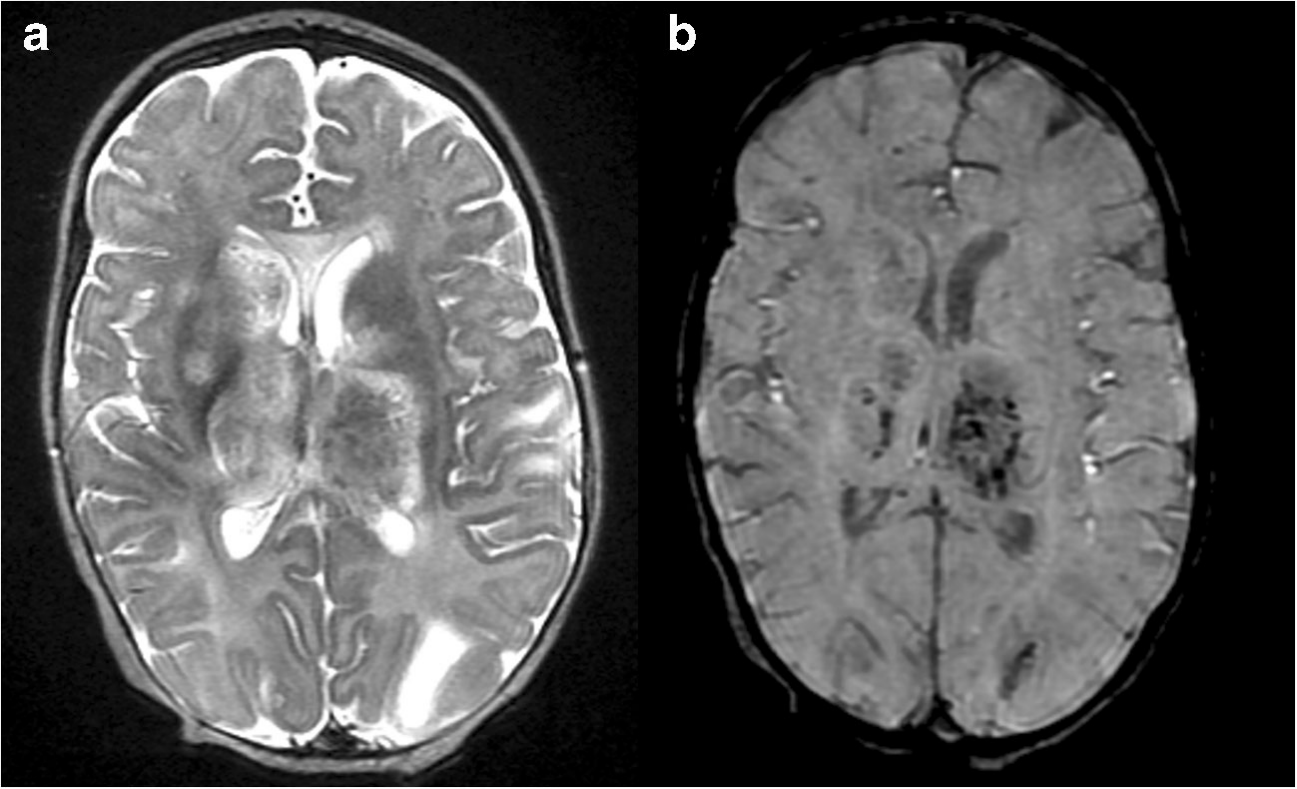
Axial T2-weighted (**a**) and SWI (**b**) MR images of the brain at the level of the basal ganglia and thalami. Extensive signal abnormalities are shown in both the basal ganglia and thalami (**a**) with hemorrhagic foci mainly located in the thalami (**b**)

**Fig. 2:**
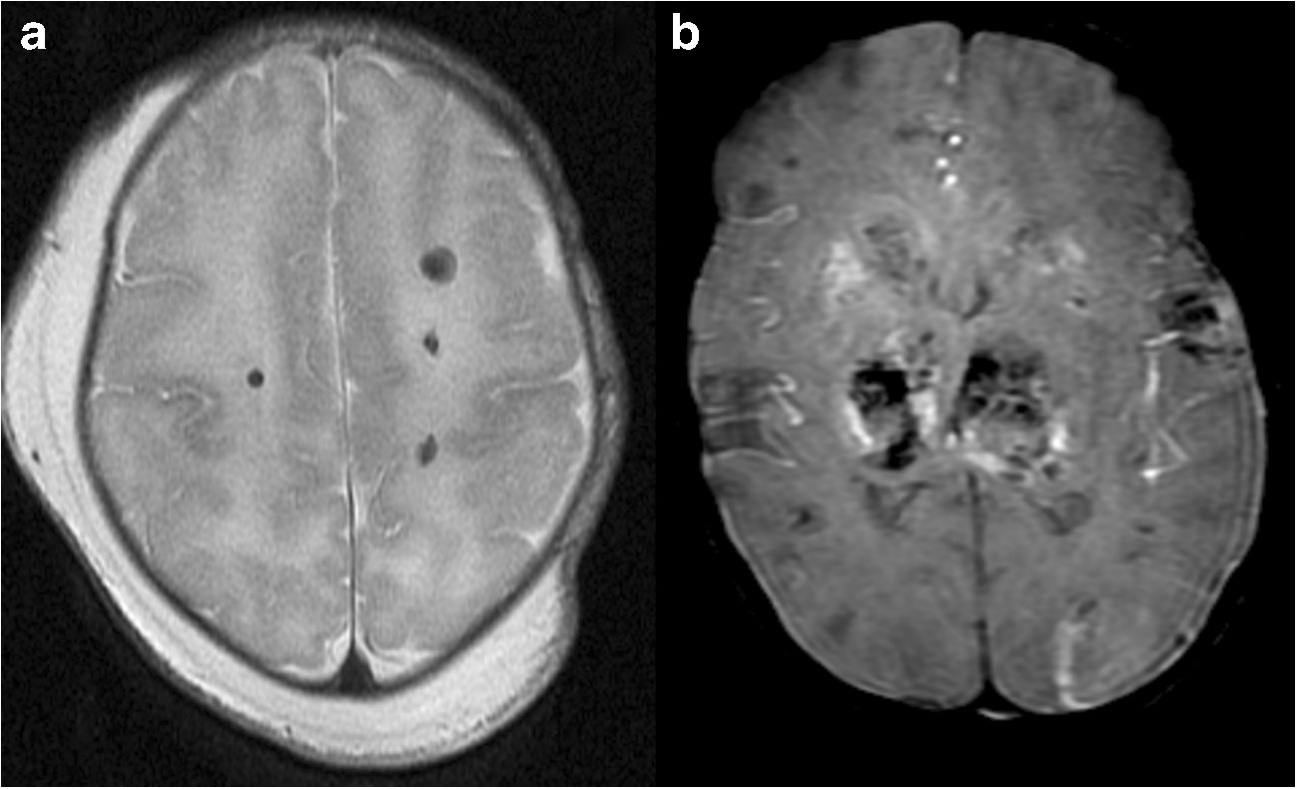
Axial T2-weighted MR image (**a**) at the level of the deep white matter and axial SWI MR image (**b**) at the level of the thalami. Focal hemorrhagic lesions are shown in the deep white matter (**a**) and more confluent hemorrhagic foci are demonstrated in both thalami (**b**)

Brain MRI was performed and demonstrated extensive asymmetric bilateral non-enhancing T2 hyperintense abnormalities with microbleeds in the basal ganglia and thalami and to a lesser extent in the cortical-subcortical region of both hemispheres and in the mesencephalon (Fig. 1). Two nodular enhancing lesions were present in the right caudate head and right thalamus. These neuroimaging features lead to acute hemorrhagic leukoencepalopathy (AHEM), (autosomal dominant) acute necrotizing encephalitis of childhood (ANEC), and viral encephalitis as differential diagnoses. Because of the predominant involvement of the basal ganglia and especially the thalami with multiple hemorrhagic lesions, HLH with CNS involvement was not considered as most likely diagnosis based on the imaging findings. However clinical findings could be consistent with HLH as she fulfilled 5 criteria. Despite treatment (methylprednisolone and etoposide) the girl deteriorated and developed irregular breathing pattern, a further decrease in consciousness and epilepsy. Follow-up MRI showed extensive progression of the lesions especially in the subcortical regions and brain stem (Fig. 1). The patient did not survive. Postmortem brain tissue study revealed extensive cavitating changes of both white and gray matter with influx of macrophages and histiocytes and lack of demonstrable micro-organisms in keeping with the suspected diagnosis of HLH with CNS involvement. Genetic tests including whole exome sequencing did not reveal abnormalities as a possible cause for primary HLH (Fig. 2).

## Case 2

A 2-day-old boy was born at 36 weeks of gestation. Caesarian section was performed because of placenta insufficiency and fetal distress in the context of maternal smoking. On the second day of life, the boy developed symptoms of sepsis caused by *Klebsiella oxytoca* grown from a central venous line. The boy developed respiratory problems, direct hyperbilirubinemia, persistent thrombocytopenia, leukopenia, anemia, acute liver failure, ascites, and subcutaneous edema. During the disease course, the patient was treated by, e.g., antibiotics, blood transfusion, and blood exchange. Increased ferritin leads to liver biopsy which was negative for neonatal hemochromatosis. Secondary HLH in the context of *Klebsiella oxytoca* septicemia was suspected; treatment with dexamethasone and etoposide was not effective. On the 20th day of life, the patient died due to multi-organ failure. Postmortem brain MRI demonstrated multiple hemorrhagic lesions in the deep gray matter structures and in the periventricular and deep white matter. The signal intensities of the hemorrhages were in keeping with early subacute degradation of blood. In the cerebellum blood products in a later subacute stage were seen.

## Discussion

Three neuropathological stages of HLH have been described and these stages are associated with the severity of disease and increased lymphocytic and histiocytic infiltration. Stage 1 disease shows leptomeningeal infiltration. Stage 2 demonstrates additional involvement of the adjacent brain parenchyma with perivascular infiltrations (as route of spread). Stage 3 consists of massive parenchymal infiltration which leads to demyelination, parenchymal necrosis, and calcification [6]. Both of our presented cases had large parenchymal lesions with hemorrhage and necrosis compatible with stage 3 HLH disease. These neuroinflammatory processes are considered to be related to systemic inflammation in HLH in which circulating cytokines cause endothelium injury in multiple organs ultimately leading to parenchyma injury and multi-organ failure [7].

The typical neuroimaging findings reported in literature are T2 hyper- or hypointense parenchymal lesions and brain atrophy in later stages. The T2 hypo-intensity of some of the lesions is assumed to be caused by calcifications. The lesions can demonstrate ring or nodular enhancement. In addition, leptomeningeal and perivascular enhancement can be seen [4, 8, 9]. Nodular or ring enhancing lesions are likely to be associated with compromised blood-brain barrier and active demyelination [2]. This is also reflected by peripheral restricted diffusion in the non-hemorrhagic parenchymal brain lesions corresponding to the enhancing part of the HLH lesions [1, 2]. This pattern of restriction helps to differentiate the HLH brain lesions from pyogenic or fungal abscess typically demonstrating central diffusion restriction. Both parenchymal atrophy and disturbances in circulation of cerebrospinal fluid (CSF) secondary to leptomeningeal infiltration lead to ventriculomegaly and sometimes subdural effusion [8] .

In the presented cases, the diffuse hemorrhagic transformation of the brain lesions, involving both basal ganglia and thalami as well as the brainstem, is in contrast to the typical imaging findings reported in literature.

In a study evaluating 46 cases of primary HLH with CNS involvement, thalamic or brain stem lesions have not been detected in any of the patients [5]. On the contrary, the presence of lesions located in the thalami or brain stem was considered useful in differentiating acute disseminated encephalomyelitis (ADEM) from HLH [4, 5]. CNS involvement predominantly presenting with thalamic and brainstem lesions is rare in HLH disease [10]. There are a few case reports describing involvement of basal ganglia and/or thalami in HLH with CNS involvement [7, 11, 12]. Basal ganglia and thalamic lesions in the setting of HLH are considered to express the more focal imaging pattern of HLH or be a part of the mixed diffuse/focal variant [12]. Abnormalities of the thalami, basal ganglia, and brainstem on neuroimaging are reported in the setting of Griscelli’s disease [13, 14], a rare autosomal recessive disorder in which HLH can develop. In the acute stage, lymphohistiocytic infiltration is thought to cause areas of inflammation/ischemia resulting in edema and swelling. In later stages calcifications are described probably as a sequel to necrosis and atrophy [13]. The more diffuse imaging pattern of HLH shows abnormalities consistent with demyelination, edema, and gliosis [12].

In addition to thalamus and basal ganglia involvement in HLH, the hemorrhagic features of the lesions are also controversial. In a limited amount of studies evaluating neuroimaging findings of patients with HLH disease and CNS involvement, hemorrhagic transformation of the parenchymal lesions in the cerebral and cerebellar white matter have been depicted and a very few are identified in the basal ganglia, but none of these multifocal hemorrhagic changes were located in the thalami [2, 7]. The hemorrhagic transformation of the brain lesions found in HLH might be due to ischemic injury and necrosis following perivascular infiltration [2, 12–14] in the setting of systemic inflammation characterized by cytokine storm and in the end multi-organ failure [7].

The differential diagnosis of bilateral hemorrhagic lesions of the central gray matter includes acute necrotizing encephalopathy (ANE), typically involving the bilateral thalami with necrosis and hemorrhage being the predominant findings. ANE is associated with respiratory viruses as causative factor, but also genetic causes are identified [15]. Acute hemorrhagic encephalomyelitis (AHEM) is a rare and severe form of ADEM in which variable involvement of the central gray matter is seen in addition to tumefactive white matter lesions. In cases of thalamic involvement this entity mimics ANE [11, 15]. Deep cerebral venous thrombosis of both internal cerebral veins can lead to hemorrhagic thalamic lesions due to venous ischemia with variable involvement of the basal ganglia. On neuroimaging thrombosis of the occluded veins is appreciated [15]. However HLH is generally not included in the differential diagnosis of hemorrhagic lesions of the basal ganglia and thalami.

In conclusion, despite the fact that neuroimaging manifestations of HLH disease are nonspecific and may overlap with many diseases such as inflammatory, infectious or infiltrative central nervous system disorders, multifocal hemorrhagic lesions involving the periventricular white matter, basal ganglia, thalami, and brainstem should suggest the possibility of HLH disease.

## Abbreviation

HLH: Hemophagocytic lymphohistiocytosis
